# Enhanced photocatalytic degradation of methylene blue using a novel counter-rotating disc reactor

**DOI:** 10.3389/fchem.2024.1335180

**Published:** 2024-02-23

**Authors:** Amir Hossein Ghasemi, Mohamad Javad Zoqi, Payam Zanganeh Ranjbar

**Affiliations:** ^1^ Department of Civil Engineering, Faculty of Engineering, University of Birjand, Birjand, Iran; ^2^ Department of Civil Engineering, Faculty of Engineering, University of Guilan, Rasht, Iran

**Keywords:** photocatalytic degradation, wastewater treatment, counter-rotational, modeling, azo dye

## Abstract

**Introduction:** This research introduces an innovative photocatalytic reactor designed to address challenges in wastewater treatment, with a focus on enhancing dye degradation and reducing Chemical Oxygen Demand (COD).

**Methods:** The reactor is designed with counter-rotational movements of discs to enhance hydrodynamics and mass transfer, along with a 3D-printed, interchangeable component system to boost efficacy. TiO_2_ nanoparticles, composed of 80% anatase and 20% rutile, are thermally immobilized on glass discs. The effectiveness of various treatment variables was assessed through a Central Composite Design (CCD), guided by a Response Surface Methodology (RSM) model.

**Results:** The RSM analysis reveals that the linear, quadratic, and interactive effects of the counter-rotational movements significantly influence the efficiency of dye and COD removal. The RSM model yields coefficients of determination (R^2^) values of 0.9758 and 0.9765 for the predictive models of dye and COD removal, respectively. Optimized parameters for dye removal include a pH of 6.05, disc rotation speed of 22.35 rpm, initial dye concentration of 3.15 × 10^−5^ M, residence time of 7.98 h, and the number of nanoparticle layers set at 3.99, resulting in 96.63% dye removal and 65.81% COD removal under optimal conditions.

**Discussion:** Notably, the reactor demonstrates potential for efficient treatment within a near-neutral pH range, which could reduce costs and resource use by eliminating the need for pH adjustments. The implementation of discs rotating in opposite directions marks a significant advancement in the process of dye removal.

## 1 Introduction

The textile industry, essential for economic growth, presents environmental challenges due to the release of dye pollutants, including the widely used Methylene blue (MB). The complex structure of this dye makes it difficult to degrade (Kubra et al., 2021). MB dye is prevalent in textiles and diverse industries like pharmaceuticals and food (Koyuncu and Kul, 2020). It is the most widely used dye in the textile industry (Arias Arias et al., 2020) and is considered the go-to colorant for clothing (Siddeeg et al., 2019). Eliminating dye pollutants from water can be difficult, and traditional treatment techniques like adsorption, flocculation, and microbial degradation are not always practical (Salman et al., 2023). While adsorption can move organics from water to the solid phase, it does not break them down (Rehan et al., 2023; Waliullah et al., 2023). Additionally, microbiological methods can take a long time to complete a treatment cycle. As a result, there is a pressing need to create effective and environmentally friendly methods to rapidly and thoroughly remove these pollutants. Many technologies have emerged for eliminating organic contaminants from water, and advanced oxidation processes (AOPs) are among the most competitive options for water treatment (Jiang et al., 2023). AOP is a sustainable approach to eliminating persistent organic pollutants, particularly dyes, by utilizing hydroxyl radicals (Iqbal et al., 2023). Unlike conventional methods, AOPs offer effective removal of persistent substances without generating additional waste, setting them apart from other treatment approaches (Vogelpohl, 2007).

The key aspect of AOPs is the generation of reactive hydroxyl radicals (OH•), which are responsible for breaking down various compounds (Rajendran et al., 2022). Specifically, when exposed to light, TiO_2_ facilitates the excitation of electrons from the valence band to the conduction band, provided that the photon energy meets or exceeds the bandgap of the semiconductor (Al-Musawi et al., 2021; Rahman et al., 2023). This leads to the formation of electrons and positive holes, initiating a series of chain reactions that drive the photocatalytic process (Vogelpohl, 2007). TiO_2_ is valued for its band position, stability, non-toxicity, affordability, and strong oxidation capabilities. It exists in three main polymorphs: rutile, anatase, and brookite. Among these, anatase is particularly notable for its large surface area, making it highly efficient in decomposing low-concentration pollutants in both air and water (Ohno et al., 2001; Zhang et al., 2014; Yunarti et al., 2022).

Several investigations have been carried out to examine the photocatalytic potential of TiO_2_ in breaking down various substances, such as azo dyes or textile industry wastewater (Lin et al., 2012; Li et al., 2015). Many previous studies could not scale up their designs for industrial purposes (Islam et al., 2021). However, 3D printing can solve this problem by producing complex designs efficiently and being eco-friendly and recyclable. The novel feature of the reactor introduced in this research is its unique design that allows the glass discs to rotate in opposite directions. Despite using only four discs, it achieves significant results, contrasting with designs requiring many more discs. This efficiency not only reduces the excessive use of nanoparticles and energy but also creates a more turbulent environment for the dye molecules, enhancing their attachment to the surface and leading to excellent removal efficiency. Interestingly, this reactor design exemplifies the innovative applications of 3D printing technology, which has been making strides across various industries, including automotive, aerospace, medical, and environmental processes. While current environmental applications of 3D printing are mostly confined to small-scale devices for sampling or separation, its potential for more significant innovations is vast. As challenges are addressed, 3D printing could profoundly impact sectors like energy, sensor technology, and sustainability, fostering reduced waste and emissions. In the field of 3D printing, different thermoplastics have unique characteristics. Polylactic Acid (PLA) is preferred for its ease of use, but it tends to be brittle and has a low service temperature. Acrylonitrile Butadiene Styrene (ABS) offers improved mechanical properties but can present challenges with adhesion. Polyethylene Terephthalate Glycol (PETG) is an emerging co-polyester, while Acrylonitrile Styrene Acrylate (ASA) shares similar mechanical properties with ABS and is known for its UV stability and weather resistance (Birosz et al., 2022). In the current study, ASA was utilized due to its distinctive features.

The creative essence of this study lies in the combination of a novel experimental method, the counter-rotating disc reactor, and the utilization of 3D-printed ASA filament. This reactor design significantly advances over existing methodologies in wastewater treatment. It features a unique component, a 3D-printed ASA filament, which enables the reactor to house four interchangeable glass discs designed for reversible rotation. This diverges from traditional methods that often rely on passive treatment techniques or require external mechanical or chemical assistance. The innovative counter-rotational movement of the reactor leverages physical dynamics to improve mixing and aeration while ensuring uniform exposure to the photocatalytic surface, leading to notably enhanced treatment outcomes. Additionally, the incorporation of thermally immobilized TiO_2_ nanoparticles on the rotating discs provides a robust and sustainable approach to pollutant degradation. This design aspect significantly enhances reactor efficiency, cost-effectiveness, and environmental sustainability. By integrating advanced materials with this novel mechanical design, the study offers a promising solution that addresses some limitations found in previous research, making it suitable for a broad spectrum of applications in industrial wastewater treatment.

In this study, a comprehensive investigation was conducted to assess the efficacy of photocatalytic dye degradation using TiO_2_ nanoparticles, with the properties of MB dye serving as a model pollutant. A novel rotating disc photocatalytic reactor (RDPR) was developed, utilizing the advantages of immobilized TiO_2_ nanoparticles. The innovative design of the reactor features rotating discs, which enhance dye degradation and eliminate the need for an external air pump. This is attributed to the inherent aeration and mixing capabilities of the discs, which result from their opposite rotation relative to each other. Additional design attributes encompass efficient UV radiation utilization, a 3D-printed interchangeable component system, and the strategic positioning of the rotating discs. The TiO_2_ nanoparticles used in this study had a unique composition of 80% anatase and 20% rutile and were thermally immobilized on glass discs. Furthermore, CCD-RSM (Central Composite Design-Response Surface Methodology) was employed not only to optimize but also to rigorously analyze the effects of various parameters, including pH, photocatalyst concentration, dye concentration, disc rotation speed, and residence time. This approach involved a comprehensive statistical treatment of the experimental data, utilizing CCD combined with RSM for optimization and Analysis of Variance (ANOVA) for in-depth statistical evaluation.

## 2 Materials and methods

### 2.1 Chemicals and reagents

In the study, TiO_2_ nanoparticles with a composition of 80% anatase and 20% rutile, a primary particle size of 21 nm, and a surface area of approximately 50 m^2^/g were procured from Degussa Co. (Germany), with a purity of 99.9%. For pH adjustment, nitric acid (HNO_3_) at 69% purity, hydrochloric acid (HCl) at 37% purity, and sodium hydroxide (NaOH) at 99.9% purity were supplied by Merck Co. (Germany). MB, serving as the model pollutant and also obtained from Merck Co. (Germany), was used with a purity of 99%. All reagents were used without further purification to ensure the integrity of the experimental results and maintain consistency throughout the study. The MB dye used in this study has its properties listed in Table 1. To ensure the homogeneity of the TiO_2_ suspension, an ultrasonic bath from Wise Clean Witeg Labortechnik was utilized. The irradiation source was UV-C radiation from three 9-W Philips Model lamps. A 12V DC motor rotated the discs, and a peristaltic pump (model PP-100 by Nogen Pars) ensured improved circulation of the suspension.

**TABLE 1:** Characteristics of methylene blue dye.

Chemical structure	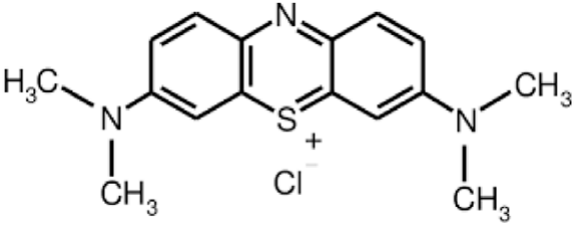
Type of dye	Cationic
Symbol	MB
Molecular formula	C_16_H_18_N_3_SCI
Molecular weight (g/mol)	319.85
Maximum absorption wavelength	640

### 2.2 Rotating disc photocatalytic reactor

**FIGURE 1:**
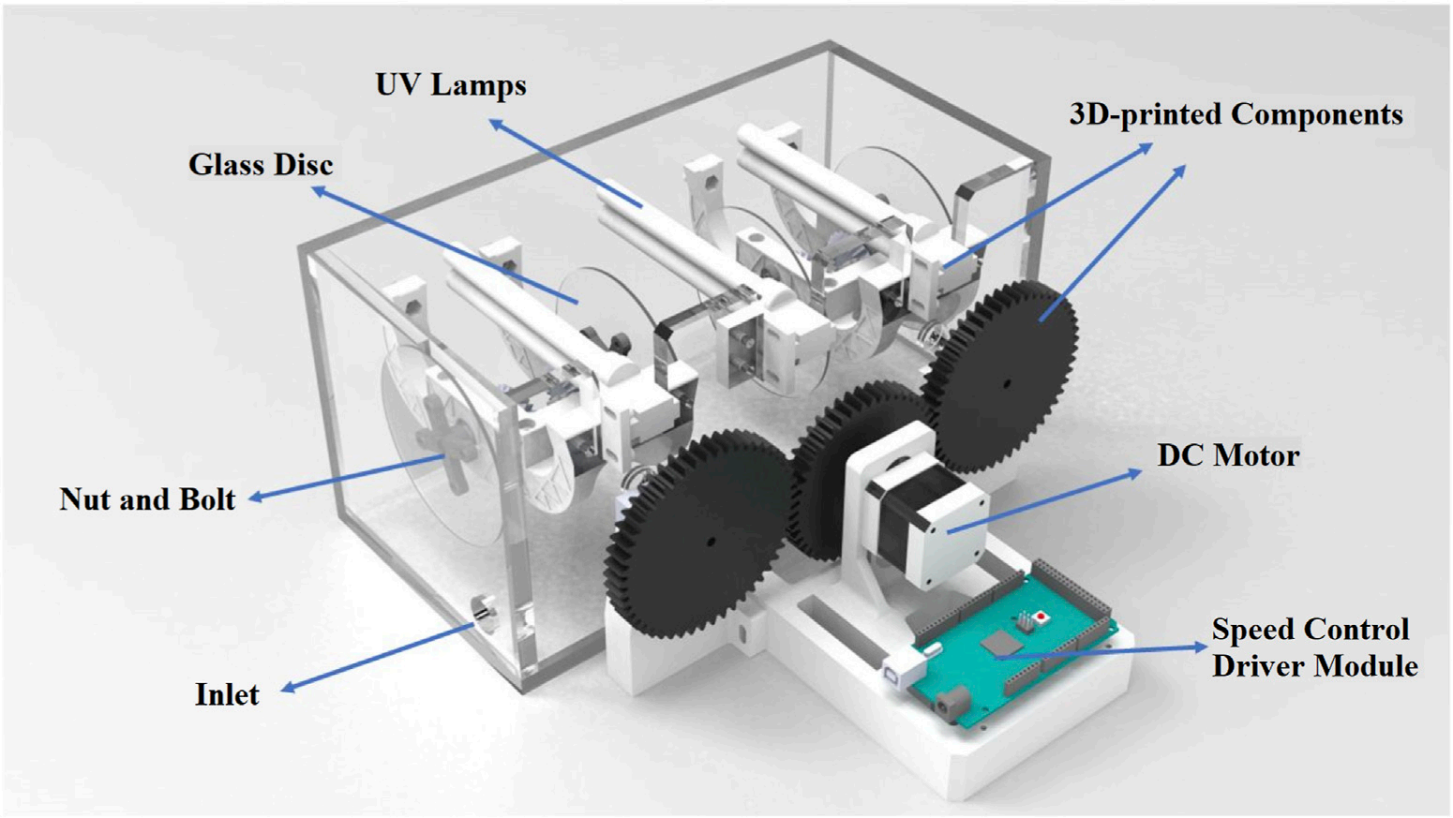
Schematic diagram of the designed reactor.

**FIGURE 2:**
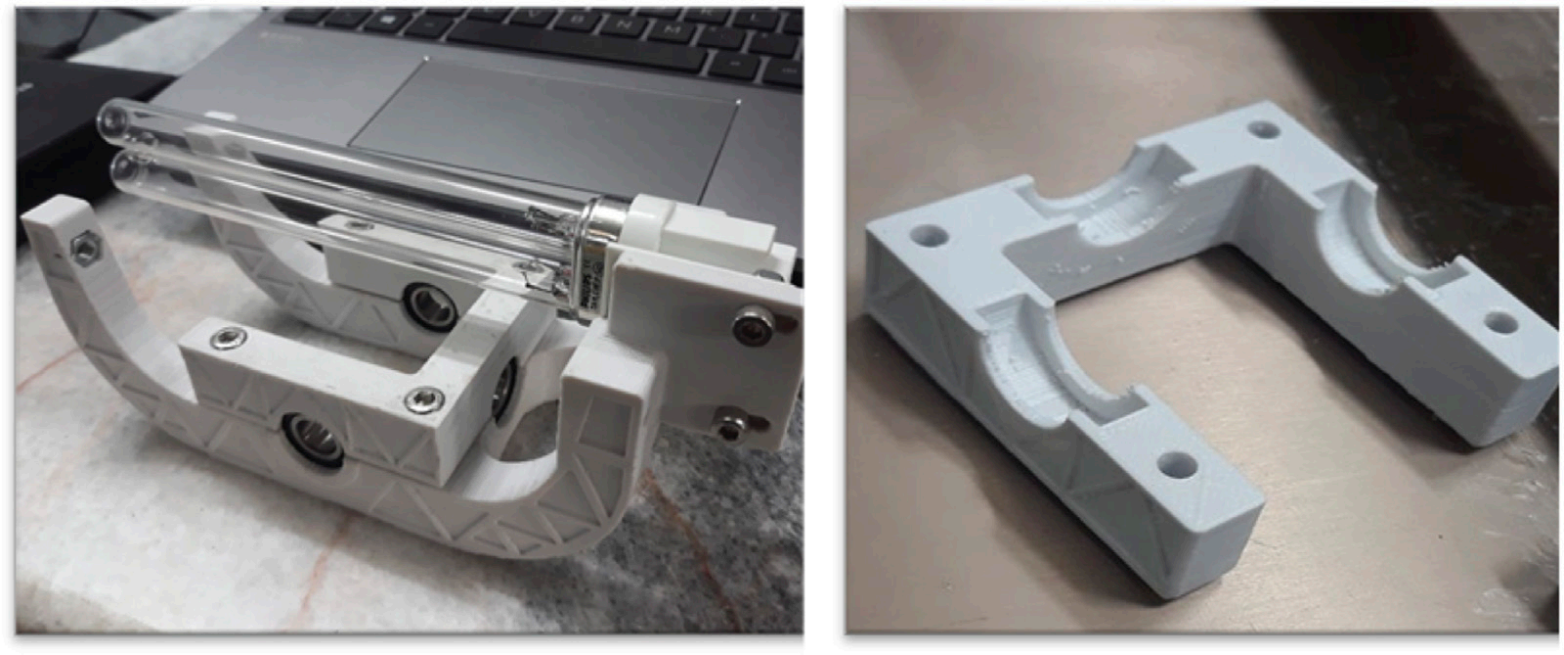
Example of a 3D-printed component.

The experimental protocols were conducted in a dedicated rectangular reactor designed specifically for synthetic wastewater, with varying MB concentrations ranging from 6.25 × 10^−6^ to 7.82 × 10^−5^ M. The reactor consisted of several sections, including a UV source, rotating glass discs coated with immobilized TiO_2_ nanoparticles, and a novel 3D-printed component, as depicted in Figure 1. A unique aspect of this reactor design is the disc rotation mechanism, where the discs rotate in opposite directions relative to each other. This feature not only facilitates enhanced aeration and mixing within the reactor but also eliminates the requirement for an external air pump. Figure 2 illustrates a sample of the 3D-printed parts. The design of the printed component allows the reactor to accommodate four interchangeable glass discs, with each disc having the capability of reversible rotation. All components produced by a 3D printer for this reactor utilized ASA filament, which has the highest resistance among all filaments against UV radiation, acidic environment, impact, and sunlight and is also recyclable. A DC motor, complemented with an adapter and drive, powers the rotation of these discs. Speed regulation is effectively managed by the integrated drive system of the motor.

The UV source is equipped with three 9 W lamps, strategically positioned to maximize the irradiation of the TiO_2_-coated discs located within the plexiglass reactor. Throughout this study, 20 glass discs were utilized. Each disc, with a thickness of 4 mm and a diameter of 11 cm, was coated with TiO_2_ nanoparticles anchored through a thermal process at varying concentrations. The effective surface area of the discs after removing the surface used for disc attachment to the reactor was 86.1 cm^2^.

In this study, a peristaltic pump was utilized to circulate the sample through a reactor made of 1 cm-thick Plexiglas. The dimensions of the reactor were 32 cm in length, 15 cm in width, and 16 cm in height, resulting in a total volume of 7.68 L. Previous research (Lin et al., 2012; Li et al., 2015), emphasized the importance of maintaining sufficient oxygen supply for AOP. This necessitated that 50% of the glass discs remain exposed above the wastewater. To meet this criterion, only 3.0 L of wastewater were utilized for each experiment, ensuring that up to 50% of the glass discs were submerged. This aligns with the design of the reactor and the RDPR system. The reactor was shielded with aluminum foil to minimize radiation dispersion and temperature control measures maintained a consistent temperature range of 23°C–24°C throughout the experimental process.

### 2.3 Immobilizing TiO_2_ nanoparticles

The immobilization of nanoparticles may be achieved through various methodologies, yet thermal immobilization is widely recognized as highly effective and efficient, as supported by prior research (Hakki et al., 2018). Thermal immobilization of nanoparticles on a substrate is a widely used method that relies on heat to establish a strong bond between the particles and the substrate, resulting in a uniform and consistent layer deposition. This technique offers several advantages, including the ability to achieve a homogeneously coated surface, ensure robust adhesion of nanoparticles, control the thickness of the deposited layer, and maintain a deposition free from contaminants (Pirsaheb et al., 2017). Moreover, the heating process can enhance specific properties of the nanoparticles, such as improved crystallinity (Tu et al., 2016). Therefore, the outstanding quality of layer deposition achieved through thermal immobilization has made it a preferred technique in numerous applications.

Prior to immobilization, the glass discs underwent a pre-treatment using a concentrated NaOH solution for 24 h to enhance their adhesive properties (Hakki et al., 2018). After this, the discs were carefully extracted from the NaOH and repeatedly rinsed with deionized water until the pH of the wash water equilibrated to 7. For the nanoparticle immobilization process, a solution was formulated by dissolving 5 g of nanoparticles in 1L of deionized water, with its pH adjusted to 3 using HNO_3_. This solution was sonicated at 50°C for 40 min in an ultrasonic bath to ensure the optimal dispersion and homogeneity of nanoparticles (Hakki et al., 2018). Using this prepared solution, one side of the glass discs was coated via a spraying method. These coated discs were then dried in an oven set at 30°C for 12 h. This coating and drying step was repeated for the opposite side of each disc. Subsequent to these coatings, the discs underwent calcination in a furnace. To prevent thermal shock during calcination, the temperature was raised incrementally, at a rate of 2°C per minute, until it reached 475°C over a period of 4 h. This temperature was then sustained for another 4 h. Following this calcination schedule, the first set of 4 discs was given a single coating. Upon extraction from the furnace, they were immediately wrapped in aluminum foil for layer protection. The described procedure was reiterated for the next set of 4 discs, which received a double coating layer. This coating procedure was consistently applied across all 20 discs. Notably, the final set of discs underwent as many as five coating iterations. Using a spray to coat the disc surface ensures a more even and uniform deposition of the layer. The weight of the glass disc was precisely 90.007 g before the TiO_2_ immobilization process. With each additional layer of TiO_2_ nanoparticles immobilization, the weight increased with exactitude to 90.021, 90.034, 90.046, 90.058, and 90.073 g for 1, 2, 3, 4 and 5 layers, respectively.

**FIGURE 3:**
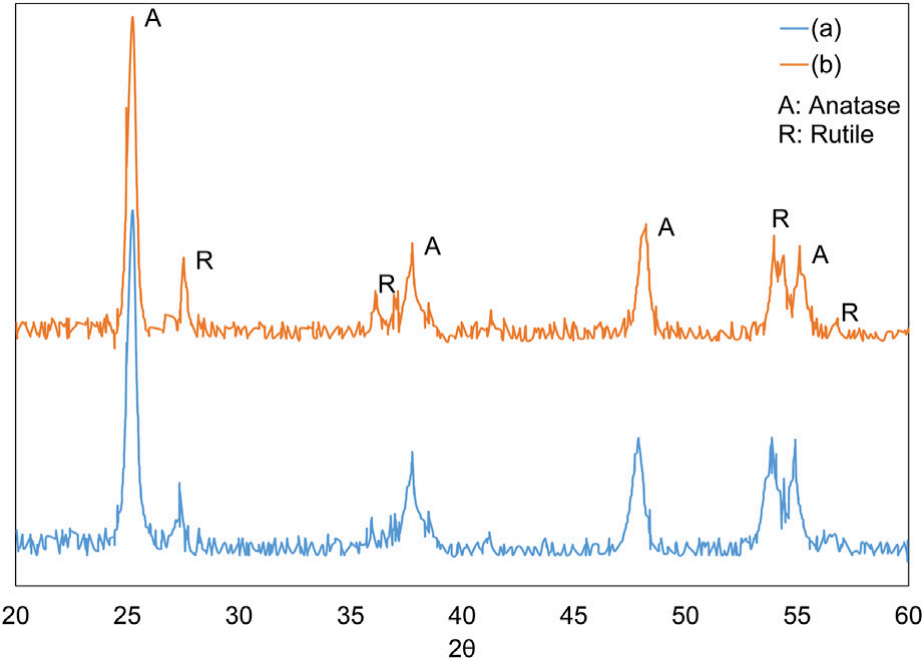
XRD pattern of **(A)** TiO_2_ and **(B)** TiO_2_ immobilized on discs at 475°C.

X-ray diffraction (XRD) analysis, conducted with a Rigaku MiniFlex 600 utilizing CuKα radiation (λ = 0.15418 nm), was used to identify the crystalline structure of the nanoparticles. Figure 3 illustrates the XRD patterns of the nanoparticles prior to and subsequent to their immobilization on the discs. Additionally, the quantification of the rutile phase present in the TiO_2_ nanoparticles, represented as XR, was conducted in accordance with Eq. 1.

$$XR=\frac{\frac{IR}{IA}\times K}{1+\frac{IR}{IA}\times K}$$ (1)

In the given equation, “IR” represents the peak height or area corresponding to the rutile phase as observed in the XRD pattern. Similarly, “IA” refers to the peak associated with the anatase phase. The constant “K” is established at a value of 0.79.

As shown in Figure 3, the increase in temperature during the thermal immobilization of nanoparticles on the disc led to a slight decrease in the anatase phase peaks and a corresponding increase in the rutile peaks. However, this change was minimal; according to Eq. 1, the anatase proportion decreased from 80% to 76%, while the rutile proportion increased from 20% to 24% as the temperature rose. These findings align with previous studies. Notably, the transformation from the anatase to the rutile phase was observed at temperatures exceeding 600°C (Machado and Santana, 2005).

The effect of the TiO_2_ immobilization process on the discs on the indirect optical band gap was investigated. The calculation of the indirect optical band gap energies for TiO_2_, both prior to and subsequent to its immobilization on the discs, was conducted using Tauc plots. The values of the band gap were ascertained through the application of Eq. 2; (Makuła et al., 2018):

$${\left(\alpha h\nu \right)}^{0.5}=B\left(h\nu -E_{g}\right)$$ (2)

**FIGURE 4:**
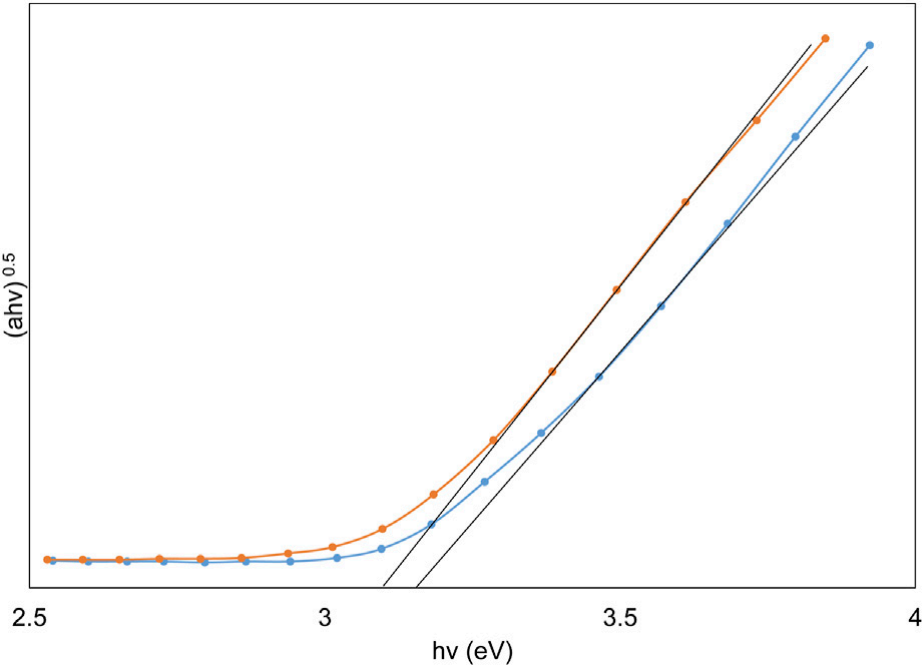
Depiction of Tauc plots highlighting the variation in band gaps of TiO_2_ nanoparticles pre and post thermal immobilization.

Wherein α denotes the absorption coefficient, hν represents photon energy, and B is a material-specific constant. The E.g., value can be deduced from the graphical representation of (αhν)^0.5^ versus photon energy (hν), achieved by extrapolating the curve until (αhν)^0.5^ approaches zero, as illustrated in Figure 4. This figure indicates that the band gap value decreases from 3.12 eV to 3.05 eV following thermal immobilization. TiO_2_ typically exists in two main phases: anatase and rutile, with the anatase phase generally having a larger band gap than the rutile phase. The XRD plot shows that during thermal immobilization, a portion of the anatase phase transforms into the rutile phase, which can lead to a decrease in the band gap. Additionally, thermal treatment can introduce defects or alter the crystallite size in TiO_2_. Smaller crystallite sizes and certain types of defects may result in quantum confinement effects or localized states in the band structure, effectively reducing the band gap (Chalastara et al., 2020).

### 2.4 Spectrophotometric analysis of MB dye and chemical oxygen demand (COD) measurement

**FIGURE 5:**
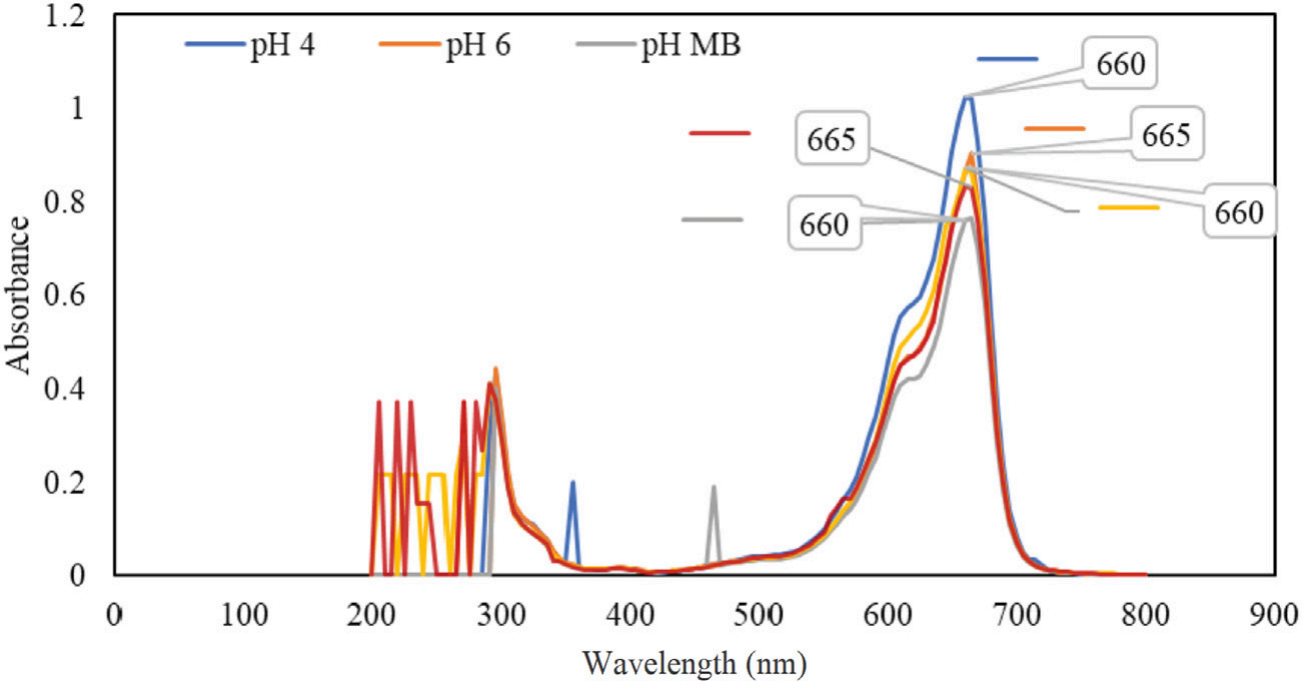
Peak absorption of MB at various pH levels.

The spectroscopic method was employed to determine the concentration of MB dye using the Beer-Lambert law. To identify the optimal wavelength for absorbance measurements, a UV/Vis Unico UV-2100 spectrophotometer was used to analyze a sample solution over a range of 200–800 nm at 5-nm intervals, as shown in Figure 5. The scan results pinpointed 665 nm as the optimal wavelength for measuring the absorbance of the solution, regardless of pollutant concentration. The wavelength was consistent with the results of other similar studies (Jassim et al., 2016; Arias Arias et al., 2020). The conversion of sample absorbance values to concentrations is achieved by using the standard curve. The standard curve is constructed by preparing multiple MB solutions with known concentrations and measuring the absorbance of each sample. The absorbance values are then plotted against their corresponding concentrations to create the standard curve.

COD was measured using a spectrophotometer device. To plot the calibration curve, stock, digestion, and catalyst solutions were first prepared. All the steps involved in this process are detailed in Method 5220 D (Rice et al., 2012).

### 2.5 Experimental design, analysis, and photocatalytic process

Among various experimental designs, the CCD is prominently adopted in many studies (Dong et al., 2009). In this study, statistical treatment of experimental data was conducted using CCD combined with RSM and evaluated using ANOVA. Additionally, CCD-RSM was employed to optimize and conduct multiple experiments for improved outcomes and cost-effective treatments. This method, complemented by ANOVA diagnostic tests, enabled comprehensive evaluation of the individual and combined effects of operational factors. These included initial pH, disc rotation speed, initial dye concentration, number of coating layers, and residence time, chosen for their significant influence on AOPs and the decolorization rate (Berkani et al., 2020; Behera et al., 2021). These variables were transformed into coded values representing five different levels, as depicted in Table 2, based on previous single-variable experiments. Following the hierarchical structure of the CCD (Gadekar and Ahammed, 2019), a total of 47 experimental runs were designed, consisting of 32 factorial, 10 star, and 5 central point runs. Each experiment was conducted three times to ensure accuracy and reliability.

**TABLE 2:** Experimental range and coded levels of independent variables.

Variables	Symbol	Coded variables level				
		−2 (-α)	−1	0	+1	+2 (+α)
Initial pH	A	2	4	6	8	10
Disc rotation speed (rpm)	B	5	15	25	35	45
Initial dye concentration (1 × 10^−5^ M)	C	1.56	3.13	4.69	6.25	7.82
Residence time (hr)	D	4	6	8	10	12
Number of coating layers	E	1	2	3	4	5

The data were regressed and visually analyzed using Design Expert 12.0 software. The degree of the model was determined at each experimental stage to find the best-fitting model. The results of the CCD were processed using RSM to fit second-order quadratic equations, allowing for the examination of the relationship between the results and operational variables. An ANOVA was performed at a 95% confidence level to identify significant effects of the variables. The adequacy of the model was confirmed by evaluating error and variance metrics, including the regression coefficient (*R* ^2^), the adjusted regression coefficient (ADJ-R^2^), and the sum of squared predicted residual discrepancies (PRESS). Once the model was validated, algorithmic optimization was conducted to determine the optimal conditions for MB dye and COD removal.

In this study, for each run, synthetic wastewater composed of distilled water and MB dye was continuously circulated within a reactor using a peristaltic pump. Glass discs coated with TiO_2_ nanoparticles were placed inside the reactor filled with a dye solution at a specific concentration. Before initiating the photocatalytic process, the discs were rotated for 30 min with the UV lamps off to establish an adsorption-desorption equilibrium. After this stage, the UVC lamps were turned on to initiate the photocatalytic process. Once the designated residence time had elapsed, the removal of dye and COD was measured. After each run, the discs were rinsed with deionized water to recover the nanoparticles and prepare them for the next experiment. To reactivate the catalyst, the disc surfaces were immersed in distilled water for 15 min and subsequently exposed to UVC lamp irradiation.

### 2.6 Field-emission scanning electron microscopy (FESEM) analysis

**FIGURE 6:**
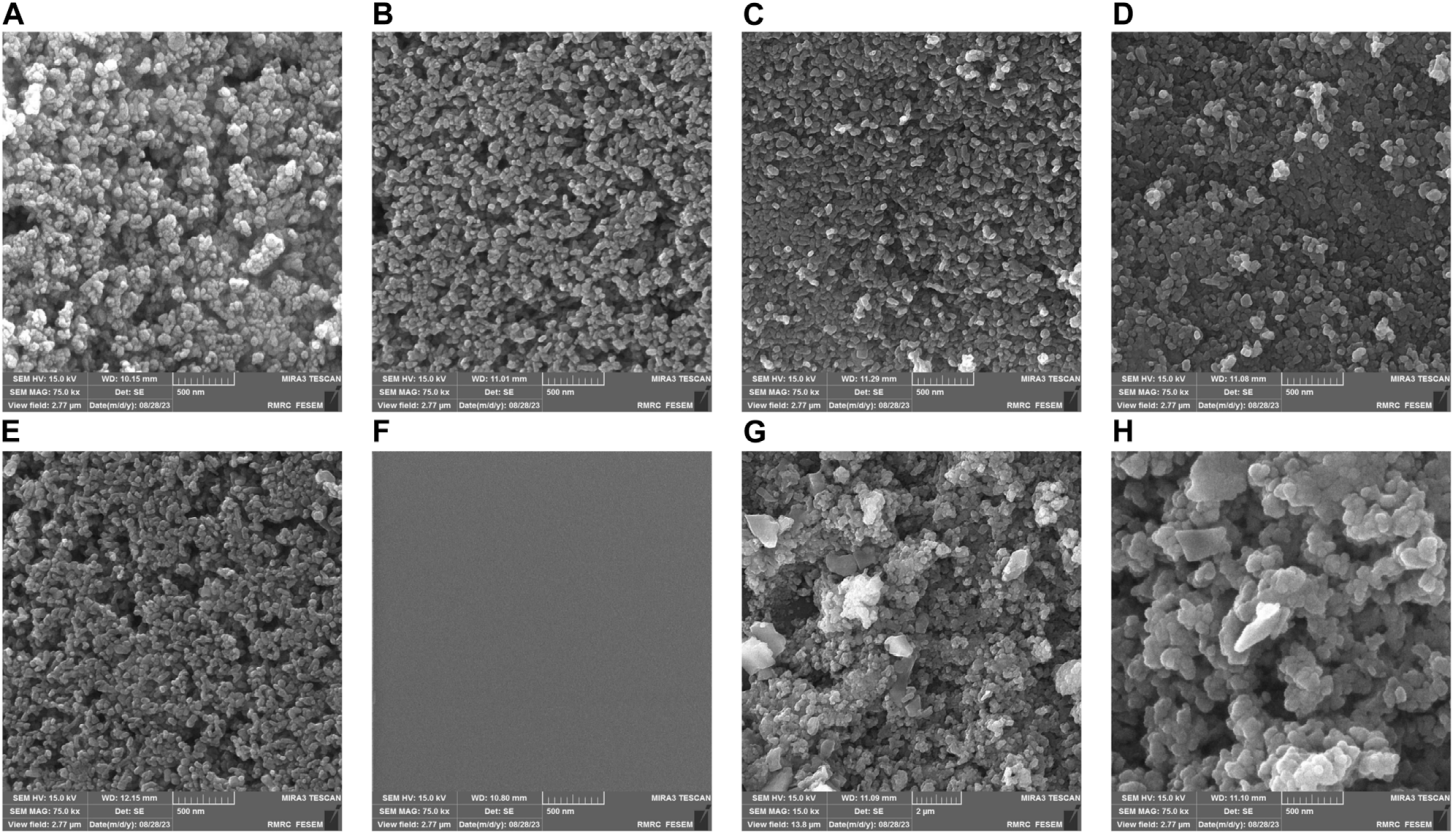
FESEM images of TiO_2_ nanoparticles coated with different layers. **(A)** 1 layer, **(B)** 2 layers, **(C)** 3 layers, **(D)** 4 layers, **(E)** 5 layers, **(F)** glass surface before coating **(G, H)** TiO_2_ nanoparticles following the experiments.

Figure 6 presents FESEM images of the discs with varying numbers of coated layers; the scale bar in the images represents 500 nm. Part (a) illustrates a disc with one layer of immobilized nanoparticles. A striking feature in this image is the voids or empty spaces between the particles. These spherical particles measure less than 50 nm. The smaller size of these particles grants them a larger specific surface area, which is advantageous for absorbing organic dyes and light. This characteristic subsequently enhances the photocatalytic activity of the coating (Hakki et al., 2018). Part (b) depicts two layers of immobilized nanoparticles. While there is an increase in the density of the particles, more space is available for additional nanoparticle fixation. In Part (c), with three layers of immobilized nanoparticles, the particle density has further increased, leaving much less space on the disc than in the previous configurations. Part (d) shows a disc with four layers of immobilized nanoparticles, where the close proximity of TiO_2_ nanoparticles to one another is evident. Part (e) displays a disc with five layers of immobilized nanoparticles. In comparison to configurations with 1–4 layers, the 5-layer structure exhibits improved uniformity. The particles are densely packed next to each other. However, there is also a noticeable clustering of particles stacked on top of one another. Given the considerations of cost and efficiency, the 4-layer structure may be the more practical choice.

Part (f) provides an image of the uncoated glass substrate. In contrast, Parts (g) and (h) display the disc after the experiments, post-immersion in MB dye. From the FESEM images in parts (g) and (h), noticeable changes were observed in the size of the thermally immobilized TiO_2_ nanoparticles after their immersion in MB. The increase in size can be attributed to the adsorption of MB molecules onto the surface of TiO_2_, resulting in a more noticeable visual representation. Moreover, the presence of MB might promote nanoparticle aggregation, even if they are thermally immobilized. Adsorbed MB molecules might bridge the gap between TiO_2_ particles, leading to their enlarged or aggregated appearance (Chin et al., 2012; Hakki et al., 2018). It is worth noting that prolonged immersion in higher MB concentrations might induce minor changes in the nanoparticle surface morphology, thereby contributing to the size variations observed (Abdellah et al., 2018).

### 2.7 Energy-dispersive X-ray (EDX) analysis

EDX is an analytical technique employed for elemental analysis or chemical characterization of a sample. In this process, a high-energy beam of charged particles, such as electrons or protons, or a beam of X-rays is directed onto the sample of interest, which in this case is the TiO_2_-coated film on the glass substrate. The elemental composition of the TiO_2_ film is presented in Table 3. In the provided EDX table, “Elt” indicates the chemical element analyzed, and “Line” refers to the characteristic X-ray emission line. “Int” represents the measured X-ray line intensity, and “Error” is its associated measurement error. The “K” and “Kr” values are ratios relating X-ray line intensities from the sample to a standard. “W%” and “A%” denote the weight and atomic percentages of each element in the sample, respectively. “ZAF” stands for corrections accounting for atomic number, absorption, and fluorescence effects. “Pk/Bg” is the signal strength relative to background noise. “Class” indicates measurement quality, with ‘A” being high quality. “LConf” and “HConf” provide confidence intervals for the measurements.

**TABLE 3:** Elemental composition of the TiO_2_-coated film on the glass substrate as determined by EDX.

Elt	Line	Int	Error	K	Kr	W%	A%	ZAF	Pk/Bg	Class	LConf	HConf
O	Ka	54.5	11.6398	0.0936	0.0576	38.71	65.41	0.1488	38.36	A	37.02	40.40
Ti	Ka	590.2	0.9043	0.9064	0.5578	61.29	34.59	0.9101	70.10	A	60.47	62.10

The weight percentage (w%) for Titanium (Ti) and Oxygen (O) are 61.29% and 38.71%, respectively. This indicates that 61.29% of the sample total weight is composed of titanium, while 38.71% is composed of oxygen. In terms of atomic percentage (Atomic%), Ti and O constitute 34.59% and 65.41% respectively, providing insight into the relative abundance of individual atoms of each element in the sample.

## 3 Results

### 3.1 RSM modeling of MB dye removal efficiency

The robust statistical methods employed in this study, including CCD, RSM, and ANOVA, played a crucial role in validating the findings. The use of CCD and RSM facilitated the comprehensive optimization and evaluation of operational factors impacting MB dye removal. The significance of these factors was rigorously assessed through ANOVA, as evidenced by the *p*-values and the fit statistics of the model (*R* ^2^, Adjusted *R* ^2^, and Adequate Precision). This statistical validation underscores the reliability of the results and the efficacy of the experimental design in determining the optimal conditions for dye removal.

In this study, a five-factor RSM was utilized to examine the effects of experimental conditions on both dye and COD removal efficiencies. Table 4 provides a brief overview of the essential findings of the study, outlining how different factors (initial pH, disc rotation speed, initial dye concentration, number of coating layers, and residence time) impact dye degradation and COD. Noteworthy trends include more effective dye removal at specific pH levels (6 and 8) and noticeable changes in response to adjustments in disc rotation speed. These selected conditions are chosen to emphasize important outcomes and provide a quick yet insightful view of how various factors influence the breakdown of dyes in wastewater treatment.

**TABLE 4:** Experimental conditions and outcomes for photocatalytic dye degradation and COD removal.

A	B	C	D	E	COD removal (%)	Dye removal (%)	Dye concentration after decolorization (1 × 10^−5^ M)
6	25	3.13	8	3	62.41	92.01	0.25
6	25	4.69	8	5	65.01	90.77	0.43
4	15	3.13	10	4	54.41	84.75	0.47
6	25	4.69	8	3	52.03	79.87	0.94
8	35	3.13	10	4	35.15	48.39	1.61
4	15	6.25	6	4	23.29	32.44	4.22
10	25	4.69	8	3	17.25	30.12	3.28
6	25	4.69	8	1	20.44	28.32	3.36
4	15	3.13	10	2	15.64	27.12	2.28
6	45	4.69	8	3	12.36	22.3	3.64
8	15	3.1	10	2	16.2	19.21	2.53
8	35	6.25	6	4	7.83	14.28	5.36

The experiments followed a CCD. Parameters with a *p*-value above 0.05 are considered statistically insignificant and can be excluded from the model unless they are necessary to maintain the hierarchy of the model (Wang et al., 2022). After removing insignificant terms, the results from the ANOVA are presented in Table 5, providing an evaluation of the adequacy of the model. Based on the ANOVA findings, the *p*-value of the model is less than 0.0001. This indicates that the quadratic model aptly describes the majority of outcomes derived from the experimental conditions suggested by the CCD (Jawad et al., 2015; Wang et al., 2022). The lack of fit of the model is statistically insignificant (0.1055 > 0.05), suggesting the compatibility of the model with the given data.

**TABLE 5:** ANOVA for the second-degree model of MB dye removal.

Source	Sum of squares	df	Mean square	F-value	*p*-value
Model	26413.41	16	1650.84	75.53	<0.0001
A	578.74	1	578.74	26.48	<0.0001
B	155.12	1	155.12	7.10	0.0123
C	5483.79	1	5483.79	250.89	<0.0001
D	906.40	1	906.40	41.47	<0.0001
E	7390.51	1	7390.51	338.12	<0.0001
AC	93.40	1	93.40	4.27	0.0474
AE	276.77	1	276.77	12.66	0.0013
BC	91.43	1	91.43	4.18	0.0497
BE	492.27	1	492.27	22.52	<0.0001
CE	574.69	1	574.69	26.29	<0.0001
DE	103.61	1	103.61	4.82	0.0473
A^2^	3317.23	1	3317.23	151.77	<0.0001
B^2^	5577.26	1	5577.26	255.16	<0.0001
C^2^	585.07	1	585.07	26.77	<0.0001
D^2^	2887.42	1	2887.42	132.10	<0.0001
E^2^	666.90	1	666.90	30.51	<0.0001
Residual	655.73	30	21.86		
Lack of Fit	629.56	26	24.21	3.70	0.1055
Pure Error	26.17	4	6.54		

Table 6 displays a strong coefficient of determination (*R* ^2^ = 0.9758), demonstrating a solid correlation between observed and predicted response values. The slight difference between the adjusted *R* ^2^ of 0.9629 and *R* ^2^ indicates that the independent variables added to the model have been selected correctly. Furthermore, a notable Adequate Precision (AP) value of 27.854, representing the signal-to-noise ratio, further confirms the appropriateness of the model. AP greater than 4 is desirable, indicating that the signal is adequately strong compared to the noise (Ghavi et al., 2021).

**TABLE 6:** Fit statistics of MB dye removal.

Std. Dev.	4.68	*R* ^2^	0.9758
Mean	39.54	Adjusted *R* ^2^	0.9629
C.V. %	11.83	Predicted *R* ^2^	0.9267
		AP	27.8544

As indicated in Table 5, the terms A, B, C, D, E, AC, AE, BC, BE, CE, DE, A^2^, B^2^, C^2^, D^2^, and E^2^ were found to be significant for the removal of MB dye. This demonstrates that all the chosen variables influence MB removal both in linear and quadratic manners. Furthermore, the interactions among the initial pH and initial dye concentration, initial pH and number of coating layers, disc rotation speed, and initial dye concentration, disc rotation speed and number of coating layers, initial dye concentration and number of coating layers, as well as residence time and number of coating layers, also have a significant effect on the response. These significant terms were incorporated into the modified model, as illustrated in Eq. 3.

$$\begin{array}{c} \text{Dye}\text{Removal}=+76.67‐3.80A‐1.97B‐11.71C+4.76D+13.59E+1.71\text{AC}‐2.94\text{AE}‐1.69\text{BC}‐3.92\text{BE}‐4.24\text{CE}\\ \;+0.3359\text{DE}‐10.65A^{2}‐13.81B^{2}‐4.47C^{2}‐9.93D^{2}‐4.77E^{2} \end{array}$$ (3)

**FIGURE 7:**
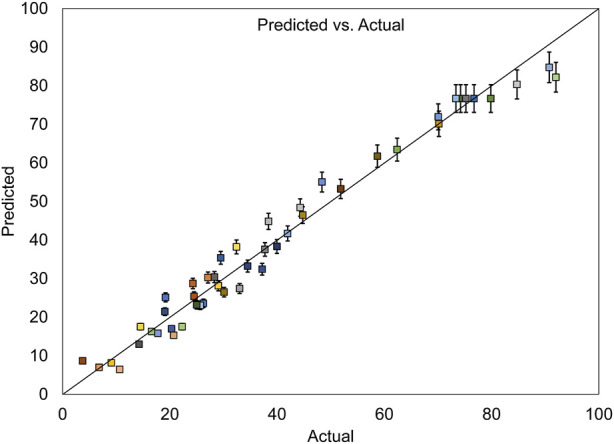
Actual and predicted values for MB dye removal, with their standard deviation error bars.

Figure 7, which depicts the evaluation of errors between actual and predicted values for dye removal, shows that the data points are approximately aligned along a straight line. This alignment affirms the desirability of the model due to its minimal errors.

#### 3.1.1 Correlation between pH and dye concentration

**FIGURE 8:**
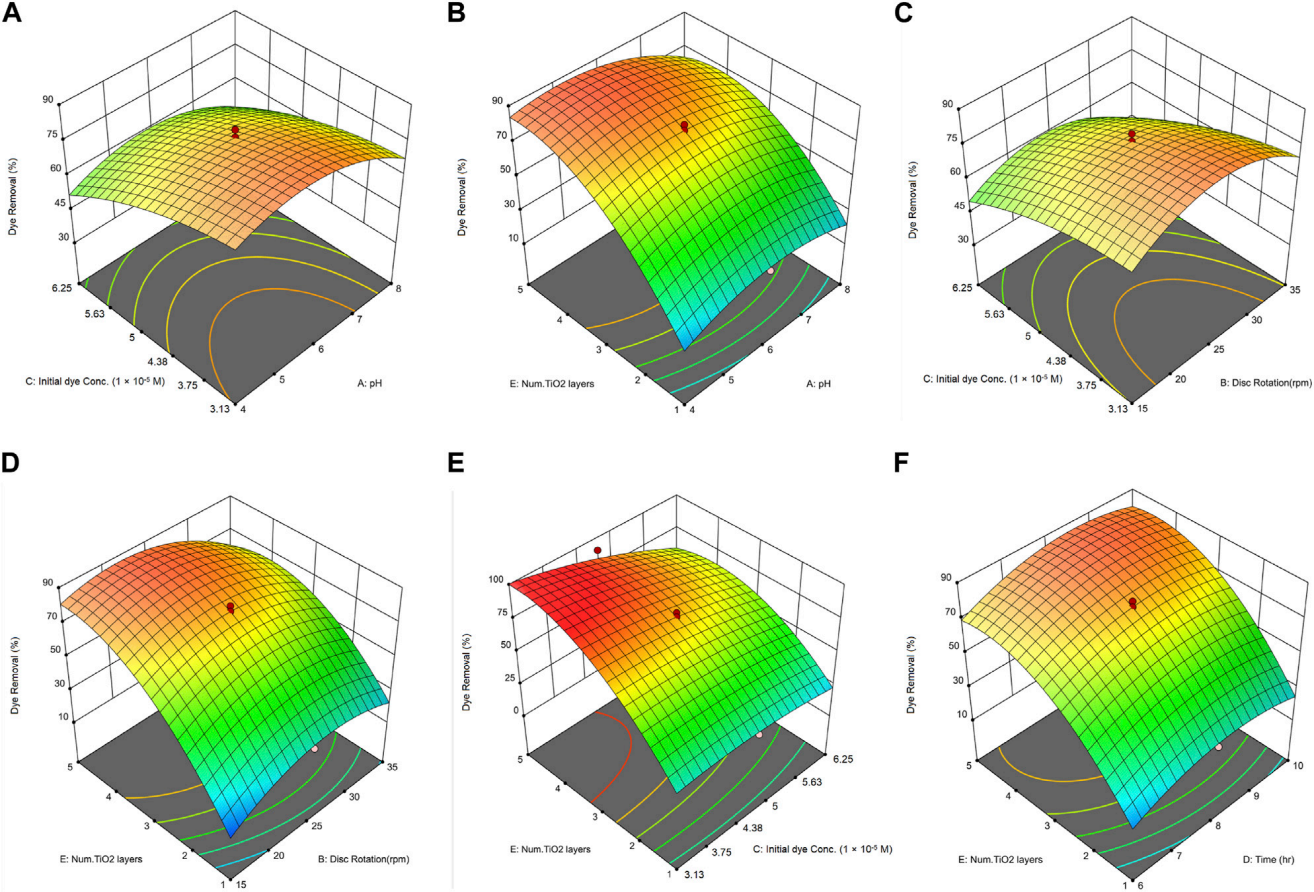
3D Surface plots illustrating the combined effects on MB dye removal of: **(A)** initial dye concentration and pH, **(B)** number of nanoparticle layers and pH, **(C)** initial dye concentration and disc rotation speed, **(D)** number of nanoparticle layers and disc rotation speed, **(E)** number of nanoparticle layers and initial dye concentration, and **(F)** number of nanoparticle layers and residence time.

In this study, the impact of five parameters on dye removal was investigated using RSM. Tests were conducted across five different factor levels. In Figure 8A, the highest removal efficiency is observed at a low MB dye concentration and a pH of 6. The efficiency decreases with an increase in dye concentration, most likely due to the competition among dye molecules for the limited active sites on the immobilized TiO_2_ nanoparticles. As the concentration of dye increases, fewer active sites are available for degradation, resulting in a reduction in overall efficiency (Berkani et al., 2020). Additionally, a dense dye concentration leads to light screening, where an excess of dye molecules absorbs or scatters UV light, hindering its penetration and subsequent interaction with the photocatalyst. This interference limits the generation of reactive oxygen species (Zazouli et al., 2017; Alkaykh et al., 2020). Therefore, reducing the initial dye concentration alleviates these inhibitory factors, enabling optimal interactions between the dye, catalyst, and UV light, thereby enhancing dye removal efficiency. This observation is consistent with previous studies. Dariani et al. (2016) noted a decrease in efficiency as dye concentration rose from 1.56 × 10^−5^ to 6.25 × 10^−5^ M. Similarly, Dai et al. (2013) found a reduction in MB removal efficiency using TiO_2_ nanoparticles when increasing the concentration from 1.56 × 10^−5^ to 9.38 × 10^−5^ M. Chin et al. (2012) also reported comparable findings, suggesting that higher dye concentrations might impede light from reaching the photocatalyst due to increased light absorption by the dye molecules.

Heterogeneous solid/liquid reactions, such as photocatalytic reactions, undergo several stages. In these reactions, dye molecules, serving as the reactants, are transported from the solution bulk to the catalytic surface. This surface acts as an adsorbent, retaining the reactants. Upon exposure to the appropriate light intensity, the degradation reaction begins, activating the catalyst. Once the reaction concludes, the products desorb from the catalyst surface and diffuse back into the solution. The pH of a solution is crucial when examining photocatalytic degradation reactions since it affects the surface charge of the TiO_2_ catalyst (Abdellah et al., 2018). The efficiency is enhanced in an acidic or near-neutral pH environment due to the increased production of hydroxyl radicals, which is more pronounced compared to an alkaline environment. When irradiated with UV light exceeding its band gap energy, TiO_2_ generates electron-hole (e^−^–h^+^) pairs (Ohno et al., 2001). Under acidic conditions, there is an abundance of protons (H^+^ ions). These protons can readily react with the photogenerated electrons, leading to a more prolific generation of hydroxyl radicals (•OH). These radicals, being potent oxidizing agents, can effectively degrade various organic contaminants, including MB, thus enhancing the dye degradation efficiency (Rideh et al., 1997). One of the primary challenges encountered in the field of photocatalysis is the undesired recombination of excited electrons and holes, which limits the efficiency of the photocatalysis process (Zazouli et al., 2017). In acidic environments, H⁺ ions act as electron acceptors, reducing this electron-hole recombination and thereby enhancing photocatalytic performance (Zhang et al., 2016; Zazouli et al., 2017). In research conducted by Yao and Wang (2010), the effect of pH on the photocatalytic removal of MB via TiO_2_ nanoparticles under UVC irradiation was explored. The most effective removal rate was observed at pH 2, followed by pH 9, with pH 7 being the least efficient. In another study by Rahimi et al. (2016), the removal of MB using TiO_2_ nanoparticles was investigated. The reduction of pH from 11 to 3 led to an increase in the removal rate from 58.5% to 98%, which aligns with the findings of this study. Rideh et al. (1997) posited that the interaction of TiO_2_ with cationic electron donors and acceptors is optimal at a pH higher than the zero-point charge (ZPC) of TiO_2_. Conversely, anionic electron donors and acceptors are favored in more acidic conditions. The surface charge behavior of the TiO_2_ catalyst is evident in both alkaline and acidic conditions, as explained below:

$$\text{TiOH}+{\text{OH}}^{‐}\;⇌{\text{TiO}}^{-}$$ (4)

$$\text{TiOH}+H^{+}⇌\;{\text{TiOH}}_{2}^{+}$$ (5)

Eq. 4 suggests that when the pH is greater than the point of zero charge (pHzpc), the dominant form of TiO_2_ is TiO¯. The zeta potential of the sample utilized in this research was reported as −25.49 mV. As highlighted, TiO_2_ holds a negative charge. As a result, the catalyst exhibits electrostatic attraction towards the positively charged MB molecules, leading to an enhanced degradation process (Koyuncu and Kul, 2020). At a pH of 9, the degradation ratio reached 33.8%, markedly surpassing the 18% observed at pH 7. Eq. 5 indicates that TiO_2_ can become protonated, forming (TiOH_2_^+^). In this protonated state, the positively charged TiO_2_ catalyst would exhibit electrostatic repulsion against other positively charged species, like MB cations (Jawad et al., 2015). However, this repulsive effect on MB adsorption is more pronounced at lower pH values. In acidic environments, electrons are drawn to the catalyst due to its positive charge. These electrons subsequently react with adsorbed oxygen molecules on the TiO_2_ surface to produce powerful oxidizing radicals such as •O_2_ and •OOH. These radicals play a dominant role in accelerating the degradation process, overshadowing the earlier discussed electrostatic repulsion (Hakki et al., 2018). Consequently, the catalytic activity of TiO_2_ is enhanced in both alkaline and acidic conditions, with the effect being more pronounced under acidic conditions.

Lakshmi et al. (1995) state that pHzpc values have been determined for various metal oxide particles. As a result of this amphoteric behavior, metal oxide surfaces primarily carry positive charges below pHzpc and negative charges above it. The pHzpc for TiO_2_ nanoparticles is found to be between 3.5 and 6.7. The maximum removal rate was observed around pH 7 in both the mentioned article and the current study. Given the positive charge on organic molecules like methylene blue (represented as MB^+^), the pH effect on the photocatalytic degradation of MB^+^ can be justified based on the electrostatic adsorption model, where cations are easily positioned in the negative sites on TiO_2_. The influence of pH on the photocatalytic degradation of cationic compounds has been elucidated using this electrostatic adsorption model (Kuźmiński et al., 2018). Furthermore, computer simulations based on this model have successfully utilized the Gouy-Chapman-Stern (GCS) approach to account for surface charge (Liu et al., 2013).

#### 3.1.2 Correlation between pH and number of nanoparticle layers

The concentration of the photocatalyst is a critical factor influencing the rate of photocatalytic reactions. Heterogeneous photocatalytic reactions typically show a direct relationship between photodegradation efficiency and catalyst loading. However, for every photocatalytic application, there is an optimal catalyst concentration to ensure efficient photon use without using excessive catalysts (Jawad et al., 2015). Overloading the system with a catalyst can lead to unintended consequences like unwanted light scattering and reduced light penetration. In the context of immobilized nanoparticles on discs, as displayed in Figure 8B, the highest efficiency was achieved between pH 4 and 6. As the number of nanoparticle layers on the discs increased from 1 to 4, there was a notable improvement in the removal efficiency of MB. However, increasing the layers from 4 to 5 showed no significant enhancement in removal efficiency. Based on these findings, discs with four layers of nanoparticles appear to provide optimal results. The ideal concentrations ensure efficient absorption of UV light by TiO_2_, producing reactive electron-hole pairs crucial for dye breakdown (Som et al., 2020). However, with more TiO_2_ layers, challenges emerge. As evident from Figures 6D, E, increasing the number of layers from 4 to 5 increases the likelihood of agglomeration in the nanoparticles. Nanoparticle agglomeration might reduce the reactive surface area and alter UV light dynamics more scattering than absorption, dampening photocatalysis (Som et al., 2020; Masouleh et al., 2022). Moreover, in higher TiO_2_ layers, nanoparticles could scavenge reactive species, leading to a decreased removal rate (Bouarioua and Zerdaoui, 2017). This finding suggests the presence of an optimal number of TiO_2_ layers, beyond which interactions between particles may hinder the effectiveness of individual particles. As a result, there is a nonlinear relationship between the concentration of nanoparticles and the efficiency of dye removal. The results obtained align with other similar studies. For instance, Bouarioua and Zerdaoui (2017) investigated the removal of Methylene Orange using TiO_2_ nanoparticles and UVC irradiation. They studied the effect of the number of immobilized nanoparticle layers on the removal efficiency, and the findings indicated that three coated layers were the optimal number of layers.

#### 3.1.3 Correlation between rotation speed and dye concentration

The rotation speed leads to suitable mixing and turbulence. This turbulence reduces mass transfer limitations due to the increased relative speed and mass transfer coefficient of the pollutant species in the liquid phase. As rotation speed increases, the relative speed also rises, leading to heightened fluctuations in the flow field. These intensified fluctuations subsequently boost turbulent kinetic energy (Najafabadi and Rashidi, 2019). Stirring speed is a crucial factor in the photocatalytic process as it plays an undeniable role in ensuring good contact between dye molecules and catalyst particles throughout the photocatalytic reaction. Based on Figure 8C, the interaction between the disc rotation speed and dye concentration can be interpreted as follows: Increasing the dye concentration, whether at low or high disc rotation speeds, leads to decreased efficiency. The optimal efficiency is achieved at a rotation speed of 25 rpm and low to medium dye concentrations of around 4.69 × 10^−5^ M. Therefore, considering the decrease in efficiency at higher speeds and for energy conservation reasons, 25 rpm can be regarded as an optimal rotation speed. The reduced efficiency at elevated speeds can be attributed to the intense turbulence in the solution, which may curtail the interaction between the dye solution and the immobilized nanoparticles on the disc. This indicates that beyond a certain speed, excessive turbulence may hinder the efficiency of dye removal.

Li et al. (2015) studied textile wastewater removal using a photocatalytic reactor, where the disc rotation speed was one of the key variables. This speed is crucial for effective mass transfer and impacts the thickness of the water layer on the surface of the disc. As the disc rotation speed increased from 5 rpm to 20 rpm, the COD concentration decreased, and then it remained relatively constant with further increases in rotation speed. Mass transfer of pollutants and oxygen onto the disc surface increased with rotation speed up to 20 rpm and reached a stable state. Energy consumption by the motor increased with higher rotation speeds, with the maximum power consumption occurring at rotation speeds of 40 rpm or higher. Considering the COD removal results and energy consumption, 20 rpm was chosen as the optimal rotation speed for subsequent experiments.

#### 3.1.4 Correlation between rotation speed and number of nanoparticle layers

The interaction between the disc rotation speed and the number of layers of immobilized nanoparticles is another significant factor. As evident from the three-dimensional plot in Figure 8D, discs with fewer layers demonstrate a very low efficiency at both low and high rotation speeds. However, there is a noticeable increase in efficiency when increasing the number of layers of immobilized nanoparticles. Based on these trends, the optimal rotation speed for the discs appears to be 25 revolutions per minute. According to the findings presented in Figure 8D, it is evident that the impact of the disc rotation speed on dye removal becomes less significant as the number of catalyst layers increases. When there is a high presence of nanoparticles, the system tends to reach saturation, making the impact of flow dynamics on dye removal efficiency less significant. The dominant factor becomes the intrinsic rate of degradation of the catalyst, along with potential reduced UV penetration due to light scattering or absorption by the dense nanoparticles (Narzary et al., 2020; Biglar et al., 2021). As a result, the contribution of flow adjustments through disc rotation becomes less important at a higher number of catalyst layers.

#### 3.1.5 Correlation between dye concentration and number of nanoparticle layers

Another significant parameter investigated is the interaction between MB concentration and the number of layers of immobilized nanoparticles. For this study, the pollutant concentration was examined within the range of 1.56 × 10^−5^ to 7.82 × 10^−5^ M. The three-dimensional plot, Figure 8E, suggests that increasing the number of immobilized nanoparticle layers on the discs while reducing pollutant concentration leads to the highest removal efficiency. Conversely, as the pollutant concentration increases and the number of nanoparticle layers decreases, there is a noticeable decline in the removal efficiency of MB. This decline in efficiency with a rise in pollutant concentration can be attributed to a greater number of adsorbed dye molecules on the photocatalyst surface, especially when the amount of catalyst and light intensity are kept constant. In such conditions, the number of electron-hole pairs generated by photoexcitation remains unchanged. This results in a reduced availability of hydroxyl radicals for dye removal, leading to decreased dye removal efficiency. To mitigate this limitation, either the light intensity must be increased or the number of adsorption sites on the catalyst surface (via a greater surface area) must be enhanced. If neither is adjusted, longer irradiation times become necessary to achieve the desired removal efficiency, potentially increasing the overall process cost (Bouarioua and Zerdaoui, 2017).

#### 3.1.6 Correlation between residence time and number of nanoparticle layers

This section investigates the relationship between residence time and the number of layers of immobilized nanoparticles. As depicted in Figure 8F, there is a noticeable rise in efficiency when the residence time increases from 6 to 8 h and the number of nanoparticle layers goes from 2 to 4. The highest efficiency is reached with a residence time of 8 h combined with three immobilized nanoparticle layers. Such an increase in efficiency, correlated with a rising number of immobilized nanoparticle layers on the discs, aligns with the findings of Lin et al. (2012). In their study on the removal of Methylene Orange using a rotating disc photocatalytic reactor, the removal efficiency increased with each additional layer of immobilized TiO_2_ nanoparticles, with the highest efficiency at four layers

In a study by Jawad et al. (2015), the removal of MB using a photocatalytic method and TiO_2_ nanoparticles was explored, and the effect of irradiation time on the removal efficiency of the pollutant was examined. The removal efficiency of the dye greatly improves with longer exposure of the TiO_2_ photocatalyst to light. Longer irradiation time implies the production of more hydroxyl radicals, which are responsible for oxidizing the MB dye molecules. UV irradiation of the TiO_2_ film leads to the generation of electron-hole pairs, and these generated electrons can react with absorbed oxygen and water molecules on the TiO_2_ surface to produce products such as O_2_^−^, HO_2_, or OH^−^.

In a separate study by Jassim et al. (2016), MB removal using a photocatalytic method with copper nanoparticles was examined. Their results indicated that the optimal removal efficiency was achieved after 5 h of irradiation.

### 3.2 RSM modeling of COD removal efficiency

After removing insignificant terms, the ANOVA results for COD removal efficiency are presented in Table 7, which assesses the adequacy of the model. According to the ANOVA findings, the *p*-value of the model is less than 0.0001, indicating that the quadratic model effectively describes the majority of outcomes resulting from the experimental conditions suggested by the CCD (Jawad et al., 2015; Wang et al., 2022). The lack of fit of the model is statistically insignificant (0.1067 > 0.05), suggesting that the model is compatible with the given data.

**TABLE 7:** ANOVA for the second-degree model of COD removal.

Source	Sum of squares	df	Mean square	F-value	*p*-value
Model	12817.89	14	915.56	94.96	<0.0001
A	153.12	1	153.12	15.88	0.0004
B	55.37	1	55.37	5.74	0.0226
C	2719.53	1	2719.53	282.05	<0.0001
D	440.90	1	440.90	45.73	<0.0001
E	3788.08	1	3788.08	392.88	<0.0001
AE	185.28	1	185.28	19.22	0.0001
BC	100.25	1	100.25	10.40	0.0029
BE	96.60	1	96.60	10.02	0.0034
CE	287.88	1	287.88	29.86	<0.0001
A^2^	1858.25	1	1858.25	192.73	<0.0001
B^2^	2465.35	1	2465.35	255.69	<0.0001
C^2^	143.65	1	143.65	14.90	0.0005
D^2^	1496.57	1	1496.57	155.22	<0.0001
E^2^	125.77	1	125.77	13.04	0.0010
Residual	308.54	32	9.64		
Lack of Fit	296.99	28	10.61	3.67	0.1067
Pure Error	11.55	4	2.89		

The fit statistics for COD removal are presented in Table 8. A comparison with Table 6 reveals that the statistical indices from RSM for both responses are closely aligned, indicating high efficiency in modeling both COD and dye removal in the photocatalytic process.

**TABLE 8:** Fit statistics of COD removal.

Std. Dev.	3.11	*R* ^2^	0.9765
Mean	25.48	Adjusted *R* ^2^	0.9662
C.V. %	12.19	Predicted *R* ^2^	0.9408
		Adeq Precision	34.0422

As indicated in Table 7, the terms A, B, C, D, E, AE, BC, BE, CE, A^2^, B^2^, C^2^, D^2^, and E^2^ were found to be significant for COD removal. These significant terms were incorporated into the modified model in terms of coded values, as illustrated in Eq. 6.

$$\text{COD}\text{Removal}=+49.81–1.96A–1.18B–8.25C+3.32D+9.73E‐2.41\text{AE}‐1.77\text{BC}‐1.74\text{BE}–3.00\text{CE}‐7.97A^{2}\;–\;9.18\;B^{2}\;–\;2.22C^{2}‐7.15D^{2}‐2.07E^{2}$$ (6)

**FIGURE 9:**
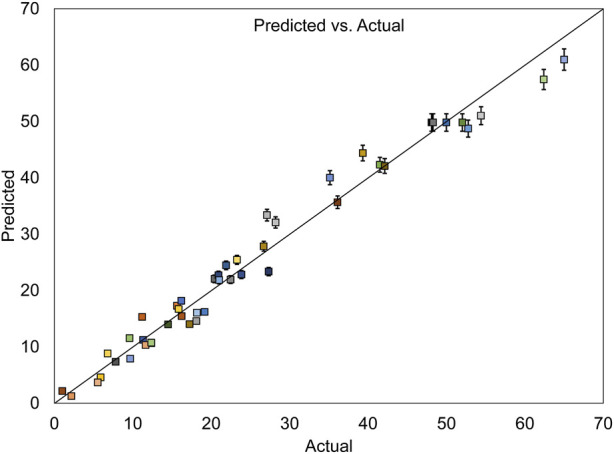
Actual and predicted values for COD removal, with their standard deviation error bars.

In Figure 9, illustrating the assessment of the error between actual and predicted values for COD removal, the data points are nearly aligned along a straight line, indicating high goodness of fit and minimal error in the model.

#### 3.2.1 Correlation between pH and number of nanoparticle layers on COD removal

**FIGURE 10:**
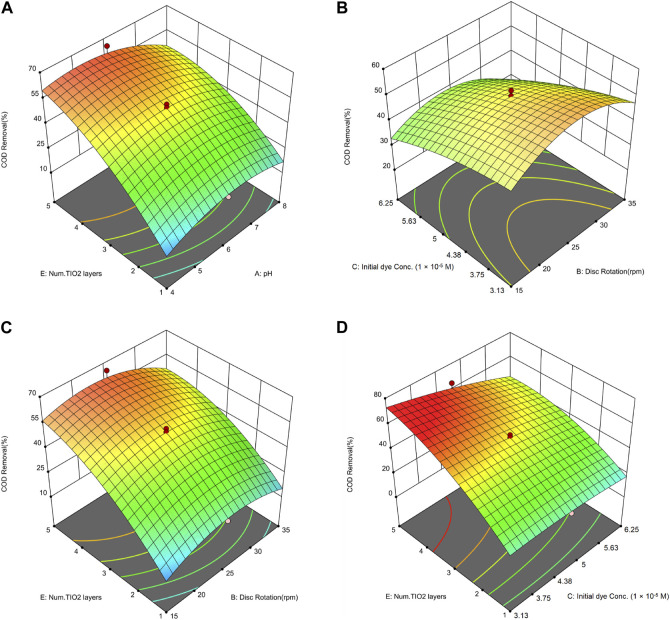
3D Surface plots illustrating the combined effects on COD removal of: **(A)** number of nanoparticle layers and pH, **(B)** initial dye concentration and disc rotation speed, **(C)** number of nanoparticle layers and disc rotation speed, and **(D)** number of nanoparticle layers and initial dye concentration.

Figure 10A highlights the important influence of the number of deposited layers of nanoparticles on COD removal, which is also observed to have a significant impact on dye removal. The effect of pH on COD removal exhibits a non-linear relationship, similar to its impact on dye removal. Specifically, increasing the pH from 4 to 6 results in an enhanced efficiency of COD removal, whereas a further increase in pH beyond 6 leads to a decline in the efficiency of COD removal. In a study conducted by Yao and Wang (2010), the efficiency of MB removal and the percentage of COD removal were examined, resulting in an efficiency of 92.3% for dye removal and 71.4% for COD removal. The highest efficiency in this study was attained under conditions of the lowest pollutant concentration (6.25 × 10^−4^ M) and a pH of 2. This finding aligns with the results of the current study, indicating that the highest efficiency is achieved in acidic environments with low pollutant concentrations.

#### 3.2.2 Correlation between rotation speed and dye concentration on COD removal

Figure 10B illustrates that analogous to the observations in dye removal, a reduction in initial dye concentration corresponds to an enhanced rate of COD removal. In a study conducted by Silvestri et al. (2020), the removal of MB was examined utilizing a photocatalytic method, aided by TiO_2_ and ZnO nanoparticles. COD served as one of the parameters to evaluate the efficacy of the AOP. The experiment involved exposing a dye solution and a photocatalyst concentration of 0.05 g/L to UV light within a reactor for a duration of 120 min. Subsequent to the photocatalytic process, the COD concentration witnessed a decline from 874 mg/L to 134 mg/L. The results of this study indicate that higher initial dye concentrations require longer exposure times in order to achieve effective removal.

In another study, Chakrabarti and Dutta (2004) investigated the removal of MB using a photocatalytic methodology, employing ZnO nanoparticles, and analyzed the impact of initial dye concentration on the degradation of MB. A decline in dye removal from 87% to 40% was observed within 2 h as the initial concentration was increased from approximately 7.82 × 10^−5^ to 3.13 × 10^−4^ M. Correspondingly, the reduction in COD removal shifted from 29% to 9.9%. The increase in initial dye concentration led to the interception of photons by entities such as dye molecules before they could interact with the catalyst surface. This resulted in a decrease in photon absorption by the catalyst.

#### 3.2.3 Correlation between rotation speed and number of nanoparticle layers on COD removal

As shown in Figure 10C, increasing the number of stabilized nanoparticle layers from 1 to 4 on the discs has significantly improved COD removal efficiency. According to Eq. 6, the relationship between the number of stabilized nanoparticle layers and COD removal exhibits a quadratic character. Assuming all other variables in Eq. 6 are held constant at level 0, the impact of varying the number of deposited layers is expressed as +9.73E—2.07 E^2^. Consequently, as the number of stabilized layers escalates, the influence of this parameter on removal efficiency diminishes. An increase in the number of deposited layers from 4 to 5 is anticipated to have a negligible impact on removal efficiency. Chakrabarti and Dutta (2004) explored the impact of nanoparticle concentration on the removal of MB and COD in their study. They discovered that elevating the nanoparticle concentration from 0.4 to 2.4 g in a 400 mL solution increased COD removal from 24% to 32%. However, further increases in nanoparticle concentration beyond 2.4 g did not enhance removal percentages. Consequently, the optimal concentration for maximal MB degradation is determined to be 2.4 g in a 400 mL solution.

#### 3.2.4 Correlation between dye concentration and number of nanoparticle layers on COD removal

The most significant interaction is evidenced between the dye concentration and the number of stabilized nanoparticle layers, as can be inferred from the F-value of this parameter (Table 7), emphasizing its importance compared to other factors. As illustrated in Figure 10D, the impact of the number of stabilized nanoparticle layers on enhancing COD removal diminishes with an increase in initial dye concentration. Furthermore, the influence of the initial dye concentration on reducing COD removal efficiency intensifies with an increase in the number of stabilized layers.

Sahoo et al. (2022) utilized a photocatalytic reactor to investigate the removal of MB from textile wastewater, focusing on the relevant parameters. COD removal for MB at flow rates of 1, 1.5, 2, and 3 L per hour was 54%, 75%, 78%, and 80%, respectively, over 120 min. COD removal decreased with an increase in initial MB dye concentration, possibly due to a decrease in the penetration depth of ultraviolet light, preventing it from reaching a greater surface area of the catalyst. With increasing dye concentration, UV absorption by dye molecules also increased. It also may be attributed to the limited availability of active sites on the photocatalyst surface for generating oxidizing species responsible for dye mineralization. COD removal for concentrations of 10, 20, and 30 ppm was 86%, 75%, and 50%, respectively, over 120 min.

Ehrampoosh et al. (2011) investigated the percentage of dye and COD removal of MB using a photocatalytic reactor with TiO_2_ nanoparticles. They achieved 98% dye removal and a simultaneous 47% reduction in initial COD. The researchers acknowledged that, generally, an increase in TiO_2_ concentration leads to an elevation in the number of active sites on the surface of the photocatalyst, subsequently augmenting the number of OH radicals and enhancing COD removal.

### 3.3 Optimization of the model for dye and COD removal

Identifying the best treatment parameters is essential to fully harness the potential of advanced treatment techniques. In this study, the objective for MB dye and COD removal was defined as maximization, and all factors and responses were assigned equal weightage. These conditions were derived through a systematic approach using the matrix method, combined with the response optimization feature in Design Expert. Referring to Eq. 3, 6, the defined optimum settings were as follows: pH at 6.05, disc rotation speed at 22.35 rpm, initial dye concentration at 3.15 × 10^−5^ M, residence time at 7.98 h, and the number of nanoparticle layers at 3.99. With these settings, a 96.63% dye removal and a 65.81% COD removal were achieved. These optimized conditions led to a significant improvement in both dye and COD removal, making them promising for practical applications. The accuracy of the predicted results was verified through the implementation of three confirmatory experiments under optimal conditions. The resulting removal percentages for the MB dye and COD were 95.12% and 64.25%, respectively, closely aligning with the predicted values. This removal efficiency was consistent with the results of other studies (Chakrabarti and Dutta, 2004; Ehrampoosh et al., 2011; Li et al., 2015; Sahoo et al., 2022).

### 3.4 Photocatalytic degradation and catalyst durability

**FIGURE 11:**
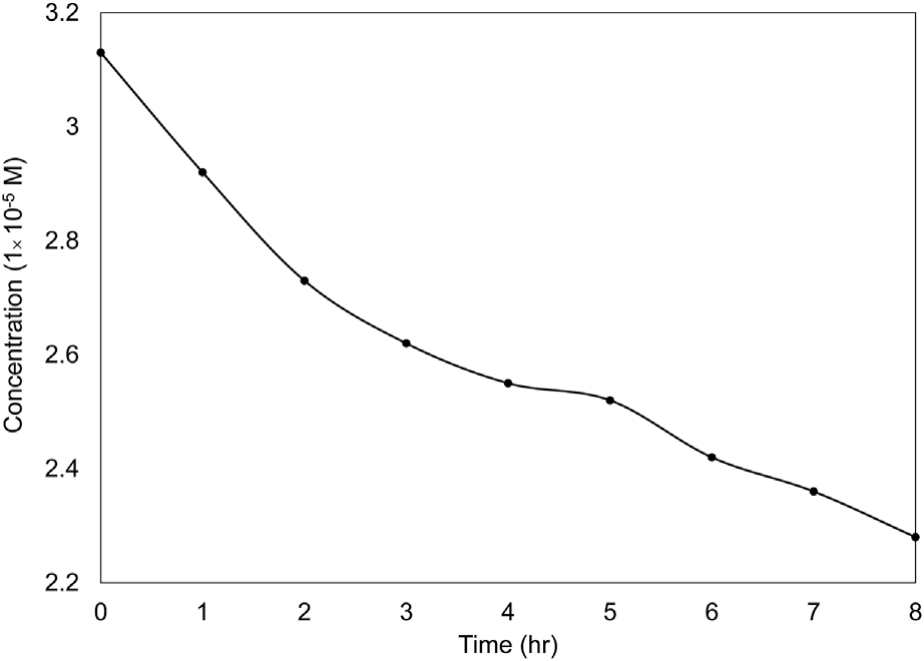
Photolysis of the dye under UVC light irradiation for 8 h in the absence of a catalyst under optimal conditions.

To investigate the effect of UVC on the dye removal process, Figure 11 illustrates the level of photodegradation under optimal conditions and in the absence of the catalyst. As depicted in this figure, in the absence of the catalyst, the dye removal rate was 27% after 8 h. The contrast between the scenarios with and without TiO_2_ nanoparticle layers emphasizes the importance of these nanoparticles as a key component in the photocatalytic degradation of dyes.

To investigate the impact of adsorption on dye removal, the wastewater was treated in the reactor without a light source (in a dark environment) under optimal conditions for 8 h. After this period, the dye removal efficiency on the nanoparticles immobilized on the discs was found to be less than 8%, which is consistent with other studies (Nguyen et al., 2018; Kurniawan et al., 2020).

In industrial settings, the repeated use of a catalyst while preserving its functional properties is crucial. This study assessed the durability of the nano-TiO_2_ catalyst for photocatalytic degradation by conducting the experiment six times under optimal conditions, using the same TiO_2_ nanoparticle-coated glass discs. The data in Table 9 reveal that the efficiency of nano-TiO_2_ in removing MB dye decreased marginally from an initial 95.1%–73.6% after six cycles. The observed reduction in dye removal efficiency can be attributed to the significant interactions occurring between the active sites of the catalyst and the dye molecules, coupled with dye adsorption (Kurniawan et al., 2020). This process results in a partial loss of functionality at some of the active sites. These results are in agreement with the findings of other studies. For instance, one study reported that after five repeated uses, the treatment effectiveness of the UV/TiO_2_ process decreased from 95% to 70% (Alvarez-Corena et al., 2016). In another study, the efficiency of dye removal using a TiO_2_ catalyst showed a decline from 99.2% to 77.4% over the course of five successive cycles (Barbosa et al., 2019). Following the sixth experiment, the TiO_2_ nanoparticle-coated glass discs were washed three times using deionized water for elution and then subjected to a 30-min ultrasound desorption process for regeneration before being reused. After the washing process, the dye removal efficiency reached 92.3%, which represents a slight decrease compared to the initial experiment cycle and is consistent with other studies (Cunha et al., 2018). This slight reduction in efficiency over successive uses highlights the substantial stability of the catalyst throughout the recycling process.

**TABLE 9:** Illustrates the effectiveness of MB dye removal across seven successive trials employing a reused nano-TiO_2_ catalyst.

Run	1	2	3	4	5	6	7
Efficiency (%)	95.1	92.4	88.3	82.2	76.4	73.6	92.3

## 4 Conclusion

In conclusion, this study introduces novel concepts in wastewater treatment, particularly through the use of a unique reactor design incorporating 3D-printed components and counter-rotating discs. This design effectively enhanced the removal of MB dye and COD, demonstrating a significant advancement over conventional methods. Findings revealed that disc rotation speed significantly impacted the removal of dye and COD. Increasing the speed of disc rotation promoted turbulence, thereby enhancing the interaction between the catalyst layer and dye molecules. Furthermore, it was observed that increasing the number of layers of immobilized nanoparticles did not significantly improve removal efficiency beyond a certain threshold. Additionally, a rise in rotational speed beyond a specific value (25 rpm) resulted in a decrease in removal efficiency, suggesting a limitation to the efficacy of augmenting nanoparticle quantities or rotational speed alone. Significantly, maintaining a near-neutral pH level in the water proved highly effective in removing dye, underscoring the fact that successful wastewater cleanup can be achieved with minimal pH adjustment requirements. A comparison with existing literature reveals that our approach, particularly in utilizing rotational dynamics and nanoparticle coatings, differs from and complements other studies. This diversity in approaches underscores the breadth of innovation in the field of wastewater treatment.

Utilizing statistical analysis techniques such as CCD, RSM, and ANOVA, the study achieves a high degree of accuracy in predicting the removal of MB dye and COD from wastewater. The application of RSM modeling yields impressive determination coefficients of 0.9758 for MB dye removal and 0.9765 for COD removal. The optimization of process conditions led to dye removal efficiency of 96.63% and COD removal efficiency of 65.81%, demonstrating the efficacy of integrating advanced reactor designs with sophisticated modeling techniques in wastewater treatment. Consequently, this study contributes significantly to the development of efficient treatment methods and lays a foundation for future research in this crucial area of environmental engineering.

## Data Availability

The original contributions presented in the study are included in the article/Supplementary Material, further inquiries can be directed to the corresponding author.
