# Supplementary figures and images for: Enhanced photocatalytic degradation of methylene blue using a novel counter-rotating disc reactor
Source: Front Chem. 2024 Feb 23;12:1335180. doi: 10.3389/fchem.2024.1335180 (PMC10920357; doi:10.3389/fchem.2024.1335180)

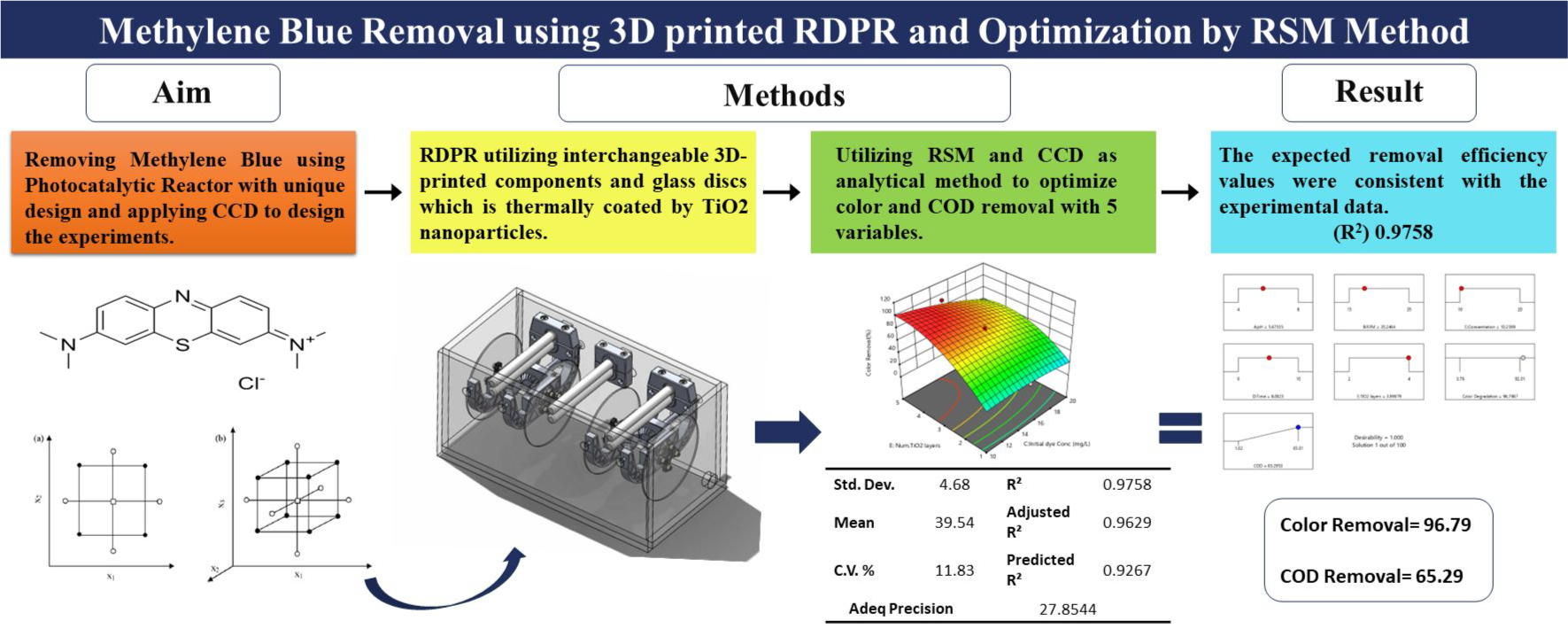

Supplement: Supplementary file 1 [file Image1.TIF]
